# Intraoperative Testing During the Mapping of the Language Cortex

**DOI:** 10.7759/cureus.36718

**Published:** 2023-03-26

**Authors:** Shabab S Kabir, Faisal R Jahangiri, Callista Rinesmith, Cristobal S Vilches, Swati Chakarvarty

**Affiliations:** 1 Neuroscience, School of Behavioral and Brain Sciences, University of Texas at Dallas, Richardson, USA; 2 Neurophysiology, Global Innervation LLC, Dallas, USA; 3 Intraoperative Neuromonitoring Program, Labouré College of Healthcare, Milton, USA; 4 Neurology, NeuroCare.AI Academy, Dallas, USA

**Keywords:** awake craniotomy, cortical stimulation, ecog, intraoperative language testing, ionm, language mapping, motor mapping, neuromonitoring, penfield, taniguchi

## Abstract

Intracranial lesions, particularly in the language-eloquent areas of the brain, can affect one's speaking ability. Despite advances in surgery, the excision of these lesions can be challenging. Intraoperative neurophysiological monitoring (IONM) during awake craniotomies can help identify language-eloquent areas and minimize postoperative impairments. Preoperative language testing is performed to establish a baseline before intraoperative language testing. This involves subjecting patients to predetermined tasks in the operating room to evaluate their phonological, semantic, and syntactic capabilities. The current state and future directions of intraoperative language testing procedures are discussed in this paper. The most common intraoperative tasks are counting and picture naming. However, some experts recommend utilizing more nuanced tasks that involve regions affected by infrequently occurring tumor patterns. Low-frequency bipolar Penfield stimulation is optimal for language mapping. Exception cases are discussed where awake craniotomies are not feasible. When dealing with multilingual patients, the patient's age of learning and skill level can be accounted for in terms of making informed task choices and mapping techniques to avoid any damage to language areas.

## Introduction

Language-eloquent tissue comprises a wide range of regions of interest, which are Broca's and Wernicke's areas. Broca's area is anatomically located in the inferior frontal gyrus (IFG) and is responsible for the motor production aspect of speech; pathological insults to this region can cause patients to be unable to generate speech but understand what is being communicated to them. On the other hand, Wernicke's area is anatomically located near the auditory cortex in the superior temporal gyrus and directly impacts language comprehension. Patients with this damaged region can still speak, although it is generally incomprehensible [1]. According to Gonen et al.'s study, Broca's region has three different neural pathways encoding phonology, semantics, and syntax relevant for testing purposes [2]. Verbal working memory is involved in the dorsal stream, while the ventral lexical-semantic network involves converting phonological sounds into meaning [3]. The aforementioned response changes are a sign of language ataxia due to damage to language areas, which includes tumors, lesions, arteriovenous malformations, aneurysms, and epilepsy foci.

The Mayo Clinic defines a brain lesion as "an abnormality seen on a brain imaging test," and they are different from normal tissue when seen on functional magnetic resonance imaging (fMRI) [4]. These lesions can result from trauma or even be acquired during surgery, such as fibrosis or scarring. Conversely, tumors are more organic and varied compared to lesions, which can be local or metastatic. Brain tumors can be classified into primary and secondary categories: primary tumors are most commonly intracranial in origin, while secondary tumors originate extracranially (breast, lung, gastrointestinal tract, and melanoma). There are two categories of tumors: benign and malignant. Malignant tumors are cancerous tumors (or tumors that invade other sites) and grow uncontrollably while potentially spreading into unrelated body areas. Malignant tumors tend to require careful treatment because of the way that they spread through the bloodstream and are more likely than benign tumors to reoccur. Glioma is a common malignant tumor. Several types include astrocytoma, oligodendroglioma, and glioblastoma, which are aggressive tumors mentioned often in the literature. There is also medulloblastoma, which is the most common malignant brain tumor in children [5]. Benign tumors stay in their primary location, so the treatment is more aggressive because of their distinct borders, and once removed, they are unlikely to return [6]. The most common benign tumor is meningioma which, while generally benign, can potentially become cancerous. While they are seen in all adults, they are slightly more common in females than in males [5].

Surgical resections of these lesions can be risky, especially due to the eloquent tissue being placed at risk of damage. Intraoperative neurophysiological monitoring (IONM) decreases said risks during awake craniotomies, specifically protecting the nervous system by providing real-time information. The introduction of IONM in surgery has positively impacted the recovery rates of patients in brain and spinal surgeries; it is said that around 53.3% of patients who had IONM intervention made complete or partial recovery of postoperative deficits by the time of discharge compared to patients requiring reoperation before discharge or experiencing little to no recovery by the time of discharge [7]. Direct cortical stimulation (DCS) is considered the gold standard for cortical mapping during awake craniotomy for delineating the boundaries of malignant tumors and accounting for individual differences between different brains. The previously collected evidence shows that DCS can accurately map the brain areas in question and identify cortical dysfunction [2]. During monitoring, the patient is asked to perform various language-based tasks that are verbal, visual, and auditory while the surgeon stimulates exposed cortical surfaces. These responses are categorized as positive and negative responses. Positive mapping signifies that the response was induced; when it cannot be induced, it is known as negative mapping [1]. Based on the responses observed, surgeons are in a more optimal position to make decisions about whether resections should be gross total or subtotal. The present work is centered around the language testing that takes place intraoperatively, aiming to summarize the recent work and future directions surrounding the process.

## Technical report

Preoperative phase

Patient Selection Based on Clinical Presentation

This research will concentrate on awake craniotomies and language cortical mapping during malignant and benign tumor resections. The inclusion criteria were based on the patient's brain tumor affecting the brain areas involved in language formation/processing [8]. Meanwhile, individuals with recurrent seizures and any form of a pacemaker or deep brain stimulation device are often excluded [8]. Males and females are, by all indications, roughly equally represented in the patients chosen.

Imaging/Investigation: Magnetic Resonance Imaging (MRI) and Diffusion Tensor Imaging (DTI)

**Figure 1:**
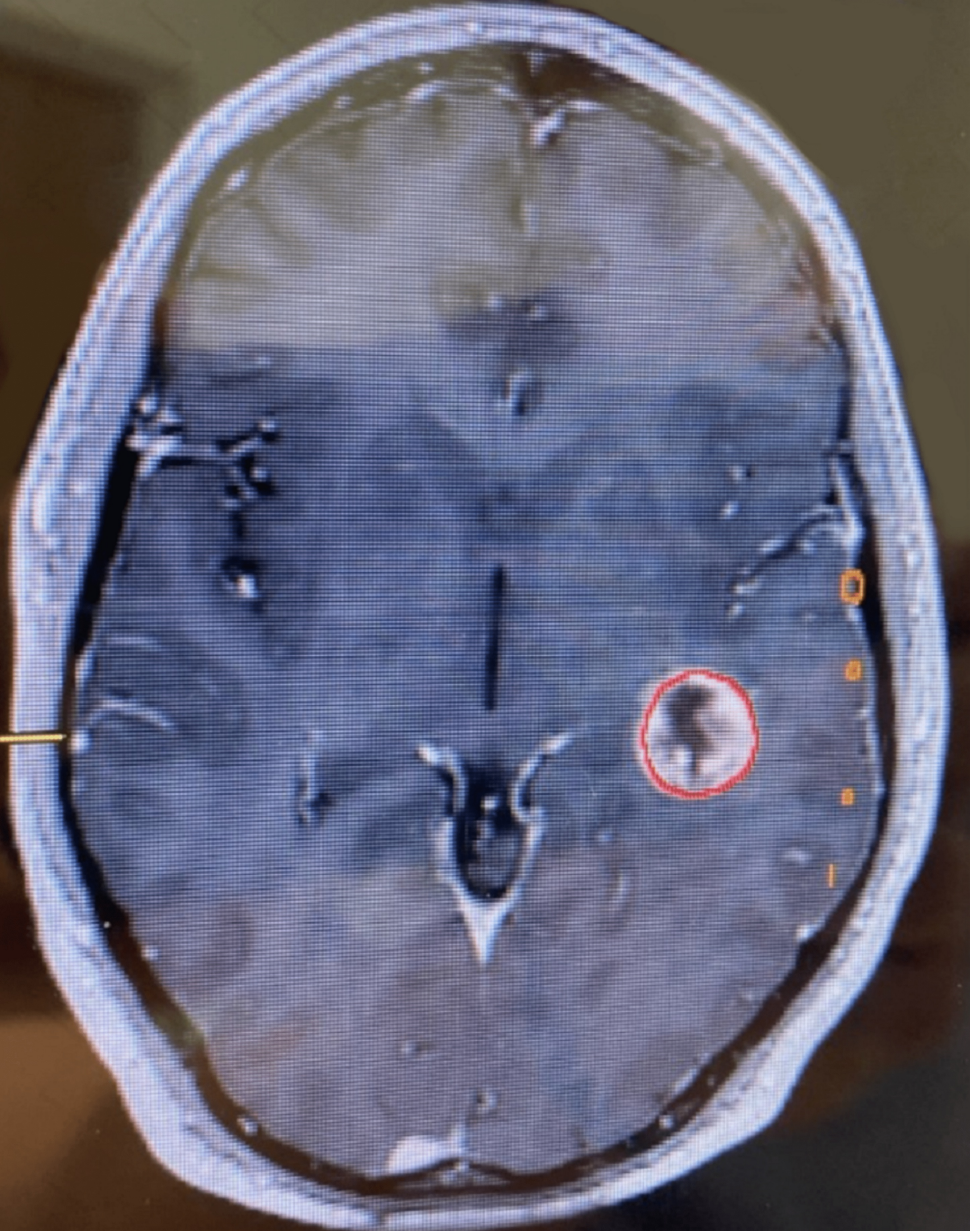
Magnetic resonance image (MRI) showing a tumor in the left cortical region.

Preoperatively, several preparations must be taken to ensure that direct cortical stimulation (DCS) and electrocorticography (ECoG) aid the surgical procedure. The first and most important step in a preoperative setting is establishing baselines for brain activity and performance by answering language-related questions. Preoperative magnetic resonance imaging (MRI), magnetoencephalography (MEG), and diffusion tensor imaging (DTI) are utilized to localize lesions. The MEG provides information regarding long-distance connectivity in language-relevant brain areas [9]. Finally, DTI visualizes the subcortical language-related axonal connections the tumor may have shifted [1]. Raffa and collaborators have shown that navigated transcranial magnetic stimulation (nTMS) can be used to pinpoint brain functional areas relevant to language skills akin to fMRI [8]. However, this practice is not considered standard yet (Figure 1).

Preoperative Language Testing: Wada, fMRI, and Clinical Testing

Functional MRI (fMRI) is utilized to localize brain functional regions related to language production and comprehension [9]. To identify the dominant language hemisphere, functional MRI provides an accurate, noninvasive alternative to the Wada test [1]. It is also crucial to note that these imaging approaches are decisively not intended to be a replacement for DCS. Giussani et al. (2009) showed that fMRI helps preoperatively. Still, studies comparing fMRI with DCS have yielded contradictory results due to inconsistencies in validation methods, differences in suitability for specific types of lesions, the feasibility of tasks that can be administered, and discrepancies in intrinsic features of both methods [10]. The anatomic location of eloquent regions may be altered by long-standing brain masses leading to differences between data from fMRI and awake mapping. Finally, all these imaging modalities require intraoperative language tasks to compare performance during the procedure. These tasks will be expanded upon in the operation section.

Electrodes

**Figure 2:**
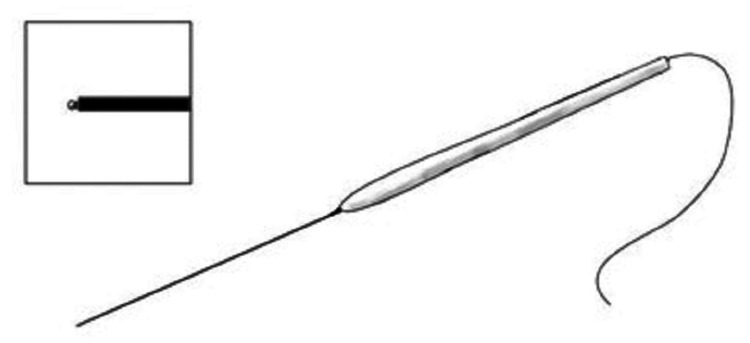
Monopolar ball tip stimulation probe. Hand-held monopolar ball tip stimulation probe used for Taniguchi method direct cortical stimulation. Source: Kabir SS, Rinesmith C, Vilches C, Chakarvarty S, Jahangiri FR: Language mapping of the brain. Introduction to neurophysiology. Jahangiri FR (ed): Kendall Hunt Publishing Company, Dubuque, IA; 2023. 303 [12]

**Figure 3:**
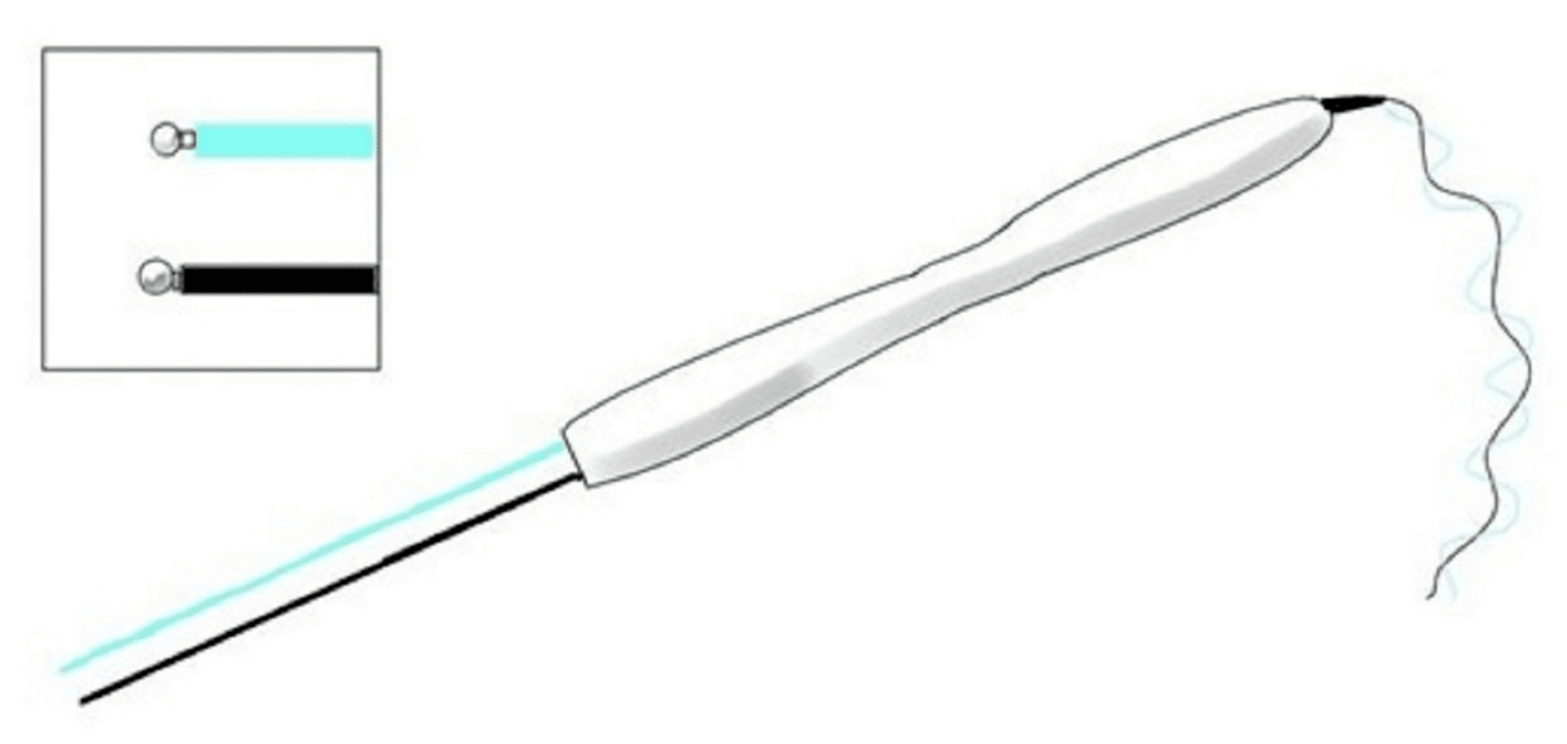
Bipolar ball tip stimulation probe. Hand-held bipolar ball tip stimulation probe used for Penfield method direct cortical stimulation. Source: Kabir SS, Rinesmith C, Vilches C, Chakarvarty S, Jahangiri FR: Language mapping of the brain. Introduction to neurophysiology. Jahangiri FR (ed): Kendall Hunt Publishing Company, Dubuque, IA; 2023. 303 [12]

**Figure 4:**
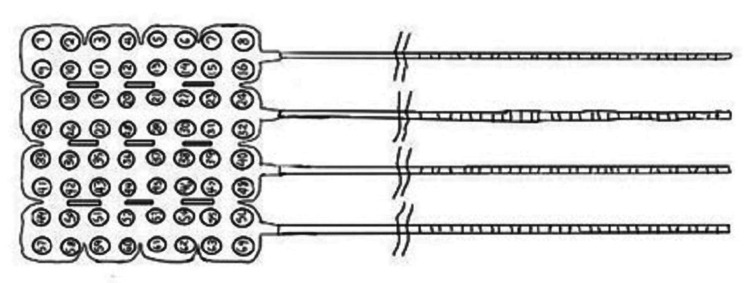
Cortical grid electrode. Schematic representations of 64-contact (8×8) cortical grid electrode. Source: Kabir SS, Rinesmith C, Vilches C, Chakarvarty S, Jahangiri FR: Language mapping of the brain. Introduction to neurophysiology. Jahangiri FR (ed): Kendall Hunt Publishing Company, Dubuque, IA; 2023. 303 [12]

Four different types of electrodes are commonly used during the linguistic cortical mapping process. These four, however, can be divided into three categories: monopolar electrodes (direct cortical motor mapping) (Figure 2), bipolar electrodes (language mapping) (Figure 3), and grid electrodes (electrocorticography) (Figure 4). Bipolar electrodes can be either subdermal needles for recordings [11] or ball tip electrodes for stimulation [1].

Intraoperative phase

Positioning

**Figure 5:**
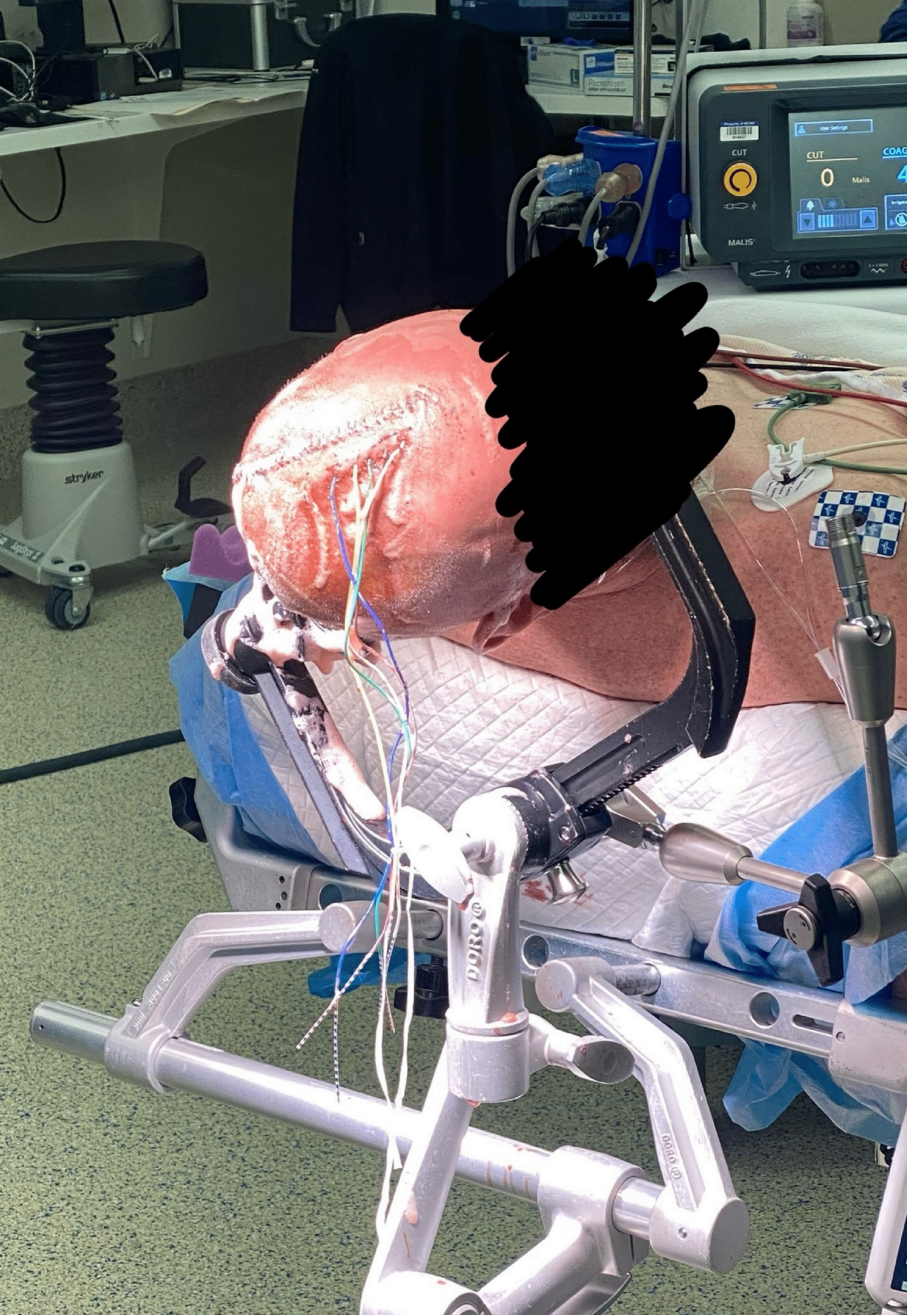
Securing the patient's head. The patient is under sedation, and the head is secured with a three-pin Mayfield holder to the operating table.

Patient positioning is the first factor to consider for a successful procedure (Figure 5). The primary and most common method is to secure the patient's head with a three-pin Mayfield holder or any modern custom holder in the lateral decubitus position (semi-lateral position) [13]. The second method is to position the patient supine while elevating the shoulder ipsilateral to the tumor and rotating the patient's head contralateral to the tumor [11]. Similarly, a holder is used in this approach to secure the patient's head.

Anesthesia

Awake craniotomy uses two types of anesthesia: asleep-awake-asleep or asleep-awake techniques. Asleep-awake-asleep is a technique that sedates the patient during the dura opening, wakes the patient for language cortical mapping, and then sedates the patient again after that process is complete. Hyperventilating the patient before dural opening helps control brain swelling in asleep-awake-asleep and asleep-awake [13]. Before dural opening, propofol is combined with an analgesic, such as fentanyl or dexmedetomidine, and administered by total intravenous anesthesia (TIVA) [11]. To stop sedation during the waking stage, the use of propofol is discontinued, while analgesics are continued [11]. Lastly, direct laryngoscopy is used to reintubate the patient to assist in a quick recovery postoperatively. An alternative may be blind nasal intubation, but this is avoided unless it is an emergency. Minors older than 10 years of age may require intermittent intravenous sedation [9].

Conscious sedation is significantly less frequently used and discussed than asleep-awake-asleep since it is more uncomfortable for the patient and has underlying complications that will be listed in the section covering the postoperative phase. Conscious sedation necessitates the administration of droperidol, propofol, or short-acting narcotics for dural opening, followed by analgesic administration until both cortical mapping and resection are complete [13]. Finally, the previously mentioned anesthetics are supplied again following resection until the procedure is completed [1].

Note that inhalants are not mentioned. The main reason is that while inhalants can be used to replace propofol during the opening of the dura, they disrupt motor evoked potential (MEP) thresholds, which can lead to postoperative impairments in the patient. As a result, their use is avoided wherever possible [14].

Direct Cortical Stimulation and Intraoperative Language Tasks

There are two common approaches to DCS/direct cortical electrical stimulation (DCES): the Penfield method and the Taniguchi method. The Penfield method is a low-frequency (50 Hz) stimulation method done via bipolar ball tip/subdermal needle electrodes in which stimulation pulse duration is between 300 and 500 µs. Stimulation is applied between three and five seconds allowing for an interstimulation interval of 5-10 seconds [1,14].

The Taniguchi method, in contrast, uses monopolar ball tip electrodes to deliver high-frequency (250-500 Hz) stimulation pulses with an approximate 500 µs stimulation pulse duration. With an average pulse duration of 500 µs, stimulation pulses are administered four to five times [1]. Starting at 2.0 mA, the intensity of these stimulations gradually increases until a positive response is obtained or until discharges are detected [1] (Tables 1-2).

**Table 1:** Stimulation parameters. Stimulation parameters for Penfield and Taniguchi methods. Source: Kabir SS, Rinesmith C, Vilches C, Chakarvarty S, Jahangiri FR: Language mapping of the brain. Introduction to neurophysiology. Jahangiri FR (ed): Kendall Hunt Publishing Company, Dubuque, IA; 2023. 305 [12]

Stimulation parameters		
Specification	Penfield	Taniguchi
Type of stimulator	Bipolar	Monopolar
Type of pulse (phase)	Biphasic or monophasic	Monophasic
Frequency	50/60 Hz	250-500 Hz
Pulse width	300-1000 µs	500 µs
Intensity	2-10 mA	2-20 mA
Duration of stimulation	2-5 s	10-20 ms

**Table 2:** Recording parameters. Recording parameters for the Penfield and Taniguchi methods. Source: Kabir SS, Rinesmith C, Vilches C, Chakarvarty S, Jahangiri FR: Language mapping of the brain. Introduction to neurophysiology. Jahangiri FR (ed): Kendall Hunt Publishing Company, Dubuque, IA; 2023. 306 [12] div, division; ECoG, electrocorticography

Recording parameters		
Specification	Penfield	Taniguchi
Low-cut filter	10 Hz	10 Hz
High-cut filter	5000 Hz	5000 Hz
Notch filter	Off for ECoG	Off for ECoG
Gain	200-500	200-500
Sensitivity	200 µV	200 µV
Time base	100 ms/div	10 ms/div

**Figure 6:**
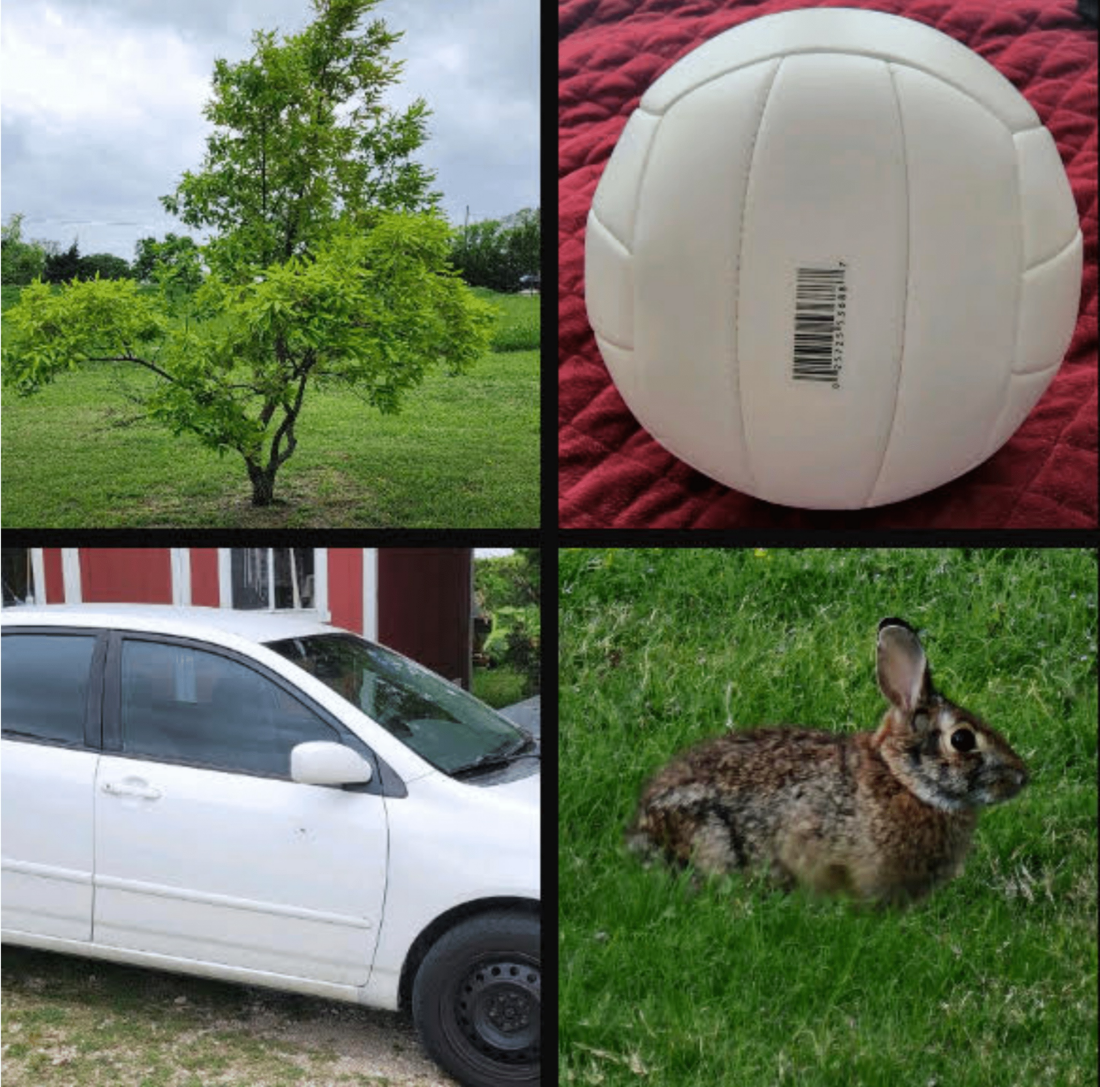
Picture naming. Picture for naming various objects (tree, volleyball, car, and rabbit).

The same array of language exercises that were completed during the preoperative stage is repeated concurrently during DCS. The discussion will center on these exercises because they are meant to assess language production and comprehension. The most frequent tasks involve counting (such as from one to 50), picture naming, reading, writing, and language syntax [9] (Figure 6) (Tables 3-7).

**Table 3:** Relatively simpler language tasks. Common language tasks in the literature. Source: Kabir SS, Rinesmith C, Vilches C, Chakarvarty S, Jahangiri FR: Language mapping of the brain. Introduction to neurophysiology. Jahangiri FR (ed): Kendall Hunt Publishing Company, Dubuque, IA; 2023. 306 [12]

Task	Brain areas	Function tapped	Errors
Picture naming: the patient identifies the target object in the image or names the target object following a verbal description	Posterior supramarginal gyrus, posterior middle temporal gyrus, and pars orbitalis	Ability to recall the appropriate word associated with target stimuli	Speech arrest, semantic paraphasias, circumlocution, phonological paraphasias, neologisms, and performance errors
Reading: the patient reads brief, unrehearsed, and unrelated sentences from a visual source	Face homunculus area, sus-sylvian postcentral gyrus, and ventral sensorimotor cortex. Superior longitudinal fascicle connecting the ventral premotor cortex with the supramarginal gyrus and the "articulatory loop"	The comprehensive interplay of phonological and semantic functions with motor, ocular, and syntactic facilities	Speech arrest and phonological or semantic paraphasias
Writing: writing outspoken text with the dominant hand and writing in a pad held up.	Exner's area, inferior parietal areas, supramarginal gyrus, and left temporoparietal cortex	Ability to internally reproduce sounds or visual representations of words	Letter omission, arrest, or illegibility
Verb generation: the patient is asked to identify semantically associated verbs, imageable, two-syllable prompts	Broca's area, other regions surrounding the perisylvian area, and posterior mid-frontal gyrus	Ability to associate semantic concepts, along with some syntax-related abilities.	Speech arrest and inability to produce a response reflecting the correct verb
Comprehension and semantic retrieval: patients are asked to use a noun or verb to fit a verbal description of an object	Posterior inferior temporal gyrus and prefrontal regions	Ability to access the correct phonological sequence to fit semantic concepts presented	Speech arrest and inability to produce a noun or verb befitting the description

**Table 4:** Speech repetition/motor-focused tasks. Tasks isolating motor components from language. Source: Kabir SS, Rinesmith C, Vilches C, Chakarvarty S, Jahangiri FR: Language mapping of the brain. Introduction to neurophysiology. Jahangiri FR (ed): Kendall Hunt Publishing Company, Dubuque, IA; 2023. 307 [12]

Task	Brain areas	Function tapped	Errors
Counting: the patient is instructed to repeatedly count from one to 10, five, or 50 slowly	Frontal motor regions, precentral gyrus, and frontal operculum	Ability to use "overlearned" language. Differentiated from areas more heavily tapping into phonological or semantic representations	Speech arrest
Verbal diadochokinesis: patients are asked to repeat the sequences of monosyllabic nonsensical words that are both similar but not identical, and sequentially identical phonemes are utilized (e.g., RAF BAF TAF versus RAF RAF RAF)	Inferior frontal gyrus (IFG), frontal ventral premotor regions, and anterior insula. Subcortically, the superior longitudinal fascicle can be targeted, responsible for linking together the supramarginal gyrus, the ventral premotor cortex, and the corticospinal tract. Uncinate fascicle	Robustness of coordination and planning in motor speech	Speech arrest and failure to maintain the alternation or repetition of phonemic rules
Repetition: the patient is familiarized with words of duo/quadrisyllabic length and easy/difficult use, along with pseudowords scrambled from existing words, which are then elicited from the patient during stimulation. Three-syllabic words and accented or consonant-clustered words are also sometimes used. A different version of this task with only real words also exists	Inferior frontal gyrus (IFG); anterior, posterior, and middle superior temporal gyrus; and supramarginal gyrus. Arcuate fasciculus connecting the inferior frontal cortex with the posterior superior temporal cortex	Ability to use phonological information from speech tracked in real time	Inability to reproduce the exact words provided and speech arrest

**Table 5:** Elimination tasks. Tasks where the patient must highlight a target response different from other options. Source: Kabir SS, Rinesmith C, Vilches C, Chakarvarty S, Jahangiri FR: Language mapping of the brain. Introduction to neurophysiology. Jahangiri FR (ed): Kendall Hunt Publishing Company, Dubuque, IA; 2023. 307 [12]

Task	Brain areas	Function tapped	Errors
Phonological odd word out: the patient is asked to read aloud one word out of four viable options per trial that do not rhyme with or otherwise share any audible features with the other three options.	Insula, inferior supramarginal gyrus, arcuate fasciculus, and inferior longitudinal fascicle	Ability to maintain phonological awareness of words accessed through lexical input methods	Speech arrest and incorrect choices
Confrontation naming: the patient is asked to identify a word or image that is not semantically associated with the three alternatives presented	Posterior/superior temporal areas, orbitofrontal and dorsolateral prefrontal areas, uncinate fascicle, inferior longitudinal fasciculus, inferior fronto-occipital fascicle	Ability to maintain the awareness of semantic relationships between concepts accessed through lexical input or visual imagery	Speech arrest and incorrect choices

**Table 6:** Tasks requiring involved patient input. Tasks seeking to tap into nuanced language abilities. Source: Kabir SS, Rinesmith C, Vilches C, Chakarvarty S, Jahangiri FR: Language mapping of the brain. Introduction to neurophysiology. Jahangiri FR (ed): Kendall Hunt Publishing Company, Dubuque, IA; 2023. 308 [12]

Task	Brain areas	Function tapped	Errors
Semantic association: patients are presented with two semantically associated but syllabically short words and asked to provide a third semantically associated word	General frontal regions and inferior fronto-occipital fascicle	Ability to tap into schematic representations and access appropriate levels of abstraction in relation to semantic themes	Speech arrest and responses that do not semantically fit the concept of the items on the prompt
Sentence completion: patients are asked to read sentences aloud and then complete them in a way that appears meaningful	Broca's area, supplemental motor area, insula, inferior longitudinal fasciculus, and subcallosal fasciculus	Ability to produce semantically and syntactically accurate speech for a particular context and dynamics between reading and language	Speech arrest and inability to complete sentences in a way that signals closure
Language syntax: the patient corrects the word order in a provided sentence with jumbled words	Inferior frontal gyrus (pars triangularis) and arcuate fasciculus	Ability to identify the accurate configuration and order of words in a sentence, reflecting a working understanding of roles being played by the words. Appears to correspond to verbal working memory function	Speech arrest and incorrect arrangement of words

**Table 7:** Language syntax tasks. The patients are tasked with arranging jumbled words into meaningful sentences: the correct order shown on the left. Source: Kabir SS, Rinesmith C, Vilches C, Chakarvarty S, Jahangiri FR: Language mapping of the brain. Introduction to neurophysiology. Jahangiri FR (ed): Kendall Hunt Publishing Company, Dubuque, IA; 2023. 309 [12]

Incorrect sentence	Correct sentence
Tree tall big stands the.	The big tree stands tall.
Very the is fast car.	The car is very fast.
Volleyball play to we like.	We like to play volleyball.
To like bunnies around hop.	Bunnies like to hop around.

Protocol for the Minors

Patients younger than 10 years of age conventionally warrant a two-stage protocol where the first stage involves subdural grid placement with language testing for the necessary highlighting of cortical areas, and the second stage entails lesion resection on a different date [9]. Minors older than 10 years of age may necessitate additional coaching throughout the process [9].

Positive/Negative Cortical Mapping, After Discharge​​​​​s (​​ADs), and Seizures

The goal of DCS approaches is to identify areas of the cortex that may result in language disruption upon stimulation (such as speech or comprehension deficits) and subsequently classify such regions as "positive" parts of the cortex while classifying unaffected regions as "negative." These areas are taped until the mapping of the language cortical regions is complete [8].

Finally, ECoG is set up with grid electrodes over the cortex to detect after discharges (ADs) and, more critically, to alert the surgical team to seizure activity. Applying ice saline to the relevant cortical areas prevents seizures [14].

Postoperative phase

After the operation, all that remains is allowing the patients to recover from anesthesia and test all neurological activity related to the regions surrounding the operated area. To achieve that goal, the patient is examined in multiple time frames, including right after the surgery, after 24 hours, after a week, after six months, and after a year [1].

## Discussion

Direct cortical electrical stimulation allows the surgical team to carry out cortical mapping to avoid postoperative deficits manifesting within the patient's life following surgery. Specifically, inadvertently caused damage to eloquent areas within language cortical areas can be circumvented and absent, in which the patient is at risk of suffering from multiple types of aphasia along with issues using language processes in tandem with other brain functions such as sensory input and motor commands. Although preoperative language testing systems alone may fulfill the need for a reference point for intraoperative language testing by characterizing the patient's baseline status [15], supplementing with the use of fMRI preoperatively or intraoperative, MRI can provide high-resolution information regarding the patient's language abilities' localization. Yet, this is not realistically feasible for every case due to the remarkable financial cost of accessing and using MRI machines. The traditional low-frequency Penfield method is utilized for DCS for language mapping due to the more appropriately prolonged stimulation duration. In contrast, the Taniguchi method's lower risk of poststimulation intraoperative seizures is more suitable for motor mapping.

Asleep-awake-asleep methods may not always be optimal for every set of circumstances, as the patient's condition in terms of age or the degree of dysfunction found may not be compatible with cortical stimulation; in these cases, asleep language mapping can be applied for regions primarily connected to Broca's area allowing the surgical team to track the integrity of motor pathways related to language use. Wada testing is also an important part of all language-related surgeries as it allows the identification of the language-dominant hemisphere for the patient. To prevent new damage incurred by the patient, ECoG is an indispensable tool as the team can be alerted to any seizure activity. Ice-cold saline can be applied immediately to avoid after discharge-related seizure activity.

While a relatively novel technique, multi-site stimulation (MSS) provides an avenue for accessing language-related regions inaccessible by DCS's single-site stimulation alone. Gonen et al. [2] found response errors they could induce in 11 out of 15 patients that did not present upon single-site stimulation. In contrast, notably, two patients did not exhibit any errors at all during single-site stimulation. The authors attribute three possible mechanical reasons for this phenomenon: firstly, the access of a third location structurally connected to the two stimulated. Secondly, the regions are connected through white matter tracts comprising greater networks, each dedicated to a key part of language function. Lastly, the sites themselves serve subfunctions in separate networks, which in turn co-activate in varying configurations to produce broader aspects of language.

**Figure 7:**
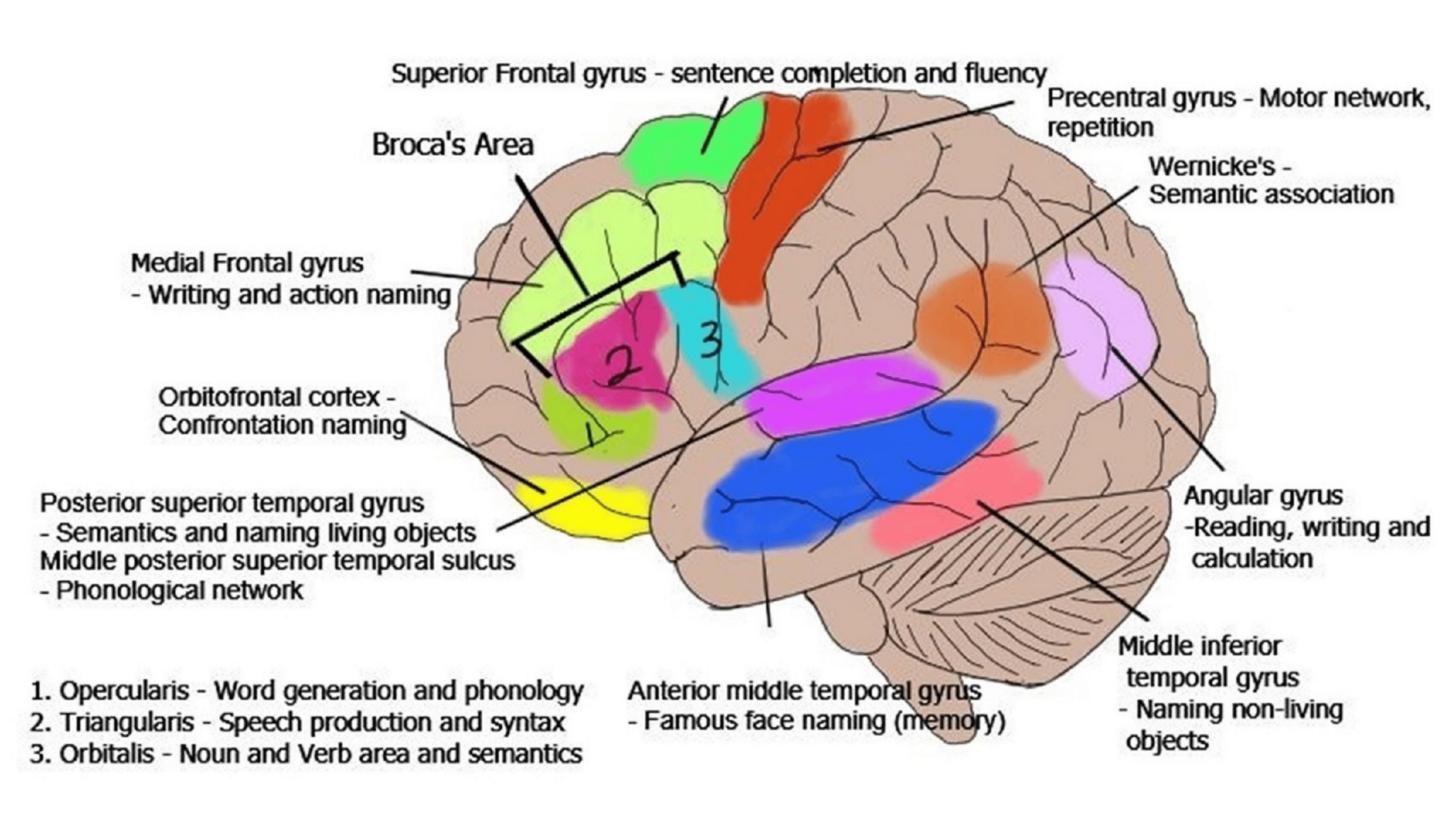
Language areas of the brain. Brain image with the consolidation of the areas involved with language. Source: Kabir SS, Rinesmith C, Vilches C, Chakarvarty S, Jahangiri FR: Language mapping of the brain. Introduction to neurophysiology. Jahangiri FR (ed): Kendall Hunt Publishing Company, Dubuque, IA; 2023. 310 [12]

Intraoperative language testing protocols have seen standardization to some degree where recent reports identify a hierarchy of tasks in terms of how commonly they are used, with picture naming ranked the highest due to how intuitive the task demands can be for the patient in addition to their universality, ease of administration, and wide applicability given the six categories of errors, with the trade-off that this task may leave some linguistic processing resources untapped. In contrast, language syntax is the most sparingly used of all tasks not only due to the inherently higher degree of involvement needed from the patient but also due to them targeting a specific subset of regions and pathways for mapping, namely, the pars opercularis and pars triangularis of the inferior frontal gyrus and the arcuate fasciculus [9]. Other tasks include counting, word repetition, reading, and writing, where brain regions such as the supramarginal gyrus, posterior/superior/anterior temporal gyri, inferior frontal, inferior parietal, basal temporal, and posterior middle temporal gyrus are involved (Figure 7).

Some of the more complex and rarely used tasks involve judging semantically incorrect sentences from correct counterparts, naming the odd phonological word/semantic concept missing, verb generation, nonsensical word generation, and rapid syllable generation/repetition [16]. These tasks represent specific targeting or isolation of functions from one another, such as planning and coordination of the motor elements of speech, separating phonological generation processes from those that involve navigating semantic concepts or for the generation of words that belong to a given category, allowing the surgical team a means to deal with cases involving irregular tumor profiles. A necessary distinction must be drawn between common tasks such as picture naming and counting and rarer tasks such as semantic association, where the former is easier to perform and track data from. Still, the latter has higher precision. It could be more suitable for the patient's assessment needs to be based on their imaging data for an approach tailored for maximal safe resections in irregular cases [17]. Therefore, these rarer high-complexity tasks allow the surgical team to preserve the function of areas more indirectly tied to language, preventing the loss of nuanced abilities utilized in conversation or abstract mental processes [16].

Regarding directions for the near future, several challenges must be tackled. Firstly, further delineation of "naming" for objects versus verbs or using visual versus auditory stimuli for descriptions is under investigation and yet to see consensus [9]. Secondly, inter-rater variability for preoperative, intraoperative, and postoperative language testing can still introduce noise sources in the data [18]. Thirdly, the patient's potential inability to answer questions due to elements such as anxiety, inattention, immaturity, and/or confusion remains a concern as the field eventually seeks to fine-tune methods differentiating such instances from the intended inability to answer questions due to stimulation [19]. Furthermore, as language regions overlap with regions involved in other cognitive functions, such as verbal working memory tapping into diverse regions such as the IFG along with the hippocampal complex or the angular gyrus with linguistic functions overlapping with calculation abilities, some authors argue in favor of expanding testing methods to accommodate tasks tapping into other cognitive abilities such as attention, executive function, nondominant hemispheric tasks such as emotion recognition, and those that recruit broader syntactic and semantic abilities [16], and it appears plausible that this could lead to further clarity in distinguishing between "critical" and "participating" brain regions [10]. Finally, the role of speech and language therapists is part of an ongoing discourse regarding capacities and functions that support the team or their training regimens [20]. It is highly recommended that only experienced team members should be involved in language cortical mapping procedures.

The population of multilingual speakers is one manifestation of the field's need for both standardization and customizing assessment processes for the individual patient. Optimally, the patient should present with no deficits in any of their spoken languages postoperatively, yet this presents a daunting challenge as most systems currently in place are geared toward monolingual individuals. There is no established method of accounting for the differences in properties between languages referred to as "distance" as it is necessary for language mapping's purposes [12]. Although an understanding of how the brain regulates mutual versus separate elements of languages is still at a relatively rudimentary stage and semantic meanings of words are thought to be represented in the brain independently from language, an idea that has gained some traction is that properties tied to specific languages tap into different cognitive resources for multilingual speakers. The age of acquisition and the degree of proficiency with which every language is spoken are considered useful metrics in this regard as a starting point.

## Conclusions

Multimodal intraoperative cortical mapping protocols have demonstrated positive outcomes allowing for optimized resection in a cost-efficient manner. We strongly recommend that the choice of intraoperative language tasks should be based on stringent preoperative clinical language testing and imaging. The other factors that should be considered may include the patient's age, number of languages known, age of acquisition, and degree of proficiency. Language mapping must be utilized by experienced teams in brain surgeries for lesions involving the language areas to minimize the incidence of postoperative neurological deficits.

## References

[1] Jahangiri FR, Chima GS, Pearson M, Jackson J, Siddiqui AA (2021). Mapping of the language cortex. Cureus.

[2] Gonen T, Gazit T, Korn A, Kirschner A, Perry D, Hendler T, Ram Z (2017). Intra-operative multi-site stimulation: expanding methodology for cortical brain mapping of language functions. PLoS One.

[3] Roger E, Banjac S, Thiebaut de Schotten M, Baciu M (2022). Missing links: the functional unification of language and memory (L∪M). Neurosci Biobehav Rev.

[4] (2022). Mayo Clinic: brain lesions. https://www.mayoclinic.org/symptoms/brain-lesions/basics/definition/sym-20050692?reDate=23022022#:%7E:text=Definition,-By%20Mayo%20Clinic&text=A%20brain%20lesion%20is%20an,look%20like%20normal%20brain%20tissue.

[5] (2022). John Hopkins Medicine: brain tumor types. https://www.hopkinsmedicine.org/health/conditions-and-diseases/brain-tumor/brain-tumor-types.

[6] Patel A (2022). Benign vs malignant tumors. JAMA Oncol.

[7] Tamkus A, Rice KS, Kim HL (2017). Intraoperative neuromonitoring alarms: relationship of the surgeon’s decision to intervene (or not) and clinical outcomes in a subset of spinal surgical patients with a new postoperative neurological deficit. Neurodiagn J.

[8] Raffa G, Bährend I, Schneider H, Faust K, Germanò A, Vajkoczy P, Picht T (2016). A novel technique for region and linguistic specific nTMs-based DTI fiber tracking of language pathways in brain tumor patients. Front Neurosci.

[9] Morshed RA, Young JS, Lee AT, Berger MS, Hervey-Jumper SL (2021). Clinical pearls and methods for intraoperative awake language mapping. Neurosurgery.

[10] Giussani C, Roux FE, Ojemann J, Sganzerla EP, Pirillo D, Papagno C (2010). Is preoperative functional magnetic resonance imaging reliable for language areas mapping in brain tumor surgery? Review of language functional magnetic resonance imaging and direct cortical stimulation correlation studies. Neurosurgery.

[11] Lu J, Wu J, Yao C (2013). Awake language mapping and 3-Tesla intraoperative MRI-guided volumetric resection for gliomas in language areas. J Clin Neurosci.

[12] Kabir SS, Rinesmith C, Vilches C, Chakarvarty S, Jahangiri FR (2023). Language mapping of the brain. Introduction to neurophysiology.

[13] Huncke K, Van de Wiele B, Fried I, Rubinstein EH (1998). The asleep-awake-asleep anesthetic technique for intraoperative language mapping. Neurosurgery.

[14] Jahangiri FR, Dobariya A, Kruse A, Kalyta O, Moorman JD (2020). Mapping of the motor cortex. Cureus.

[15] Bilotta F, Stazi E, Titi L, Lalli D, Delfini R, Santoro A, Rosa G (2014). Diagnostic work up for language testing in patients undergoing awake craniotomy for brain lesions in language areas. Br J Neurosurg.

[16] De Witte E, Satoer D, Robert E, Colle H, Verheyen S, Visch-Brink E, Mariën P (2015). The Dutch Linguistic Intraoperative Protocol: a valid linguistic approach to awake brain surgery. Brain Lang.

[17] De Witte E, Mariën P (2013). The neurolinguistic approach to awake surgery reviewed. Clin Neurol Neurosurg.

[18] Sefcikova V, Sporrer JK, Ekert JO, Kirkman MA, Samandouras G (2020). High interrater variability in intraoperative language testing and interpretation in awake brain mapping among neurosurgeons or neuropsychologists: an emerging need for standardization. World Neurosurg.

[19] Lee GP, Loring DW, Smith JR, Lee MR (2000). Predictors of patient inability to cooperate during intraoperative language mapping. Epilepsy Behav.

[20] O'Neill M, Henderson M, Duffy OM, Kernohan WG (2020). The emerging contribution of speech and language therapists in awake craniotomy: a national survey of their roles, practices and perceptions. Int J Lang Commun Disord.

