# Correction: Intraoperative Testing During the Mapping of the Language Cortex

**DOI:** 10.7759/cureus.c299

**Published:** 2025-09-16

**Authors:** Shabab S Kabir, Faisal R Jahangiri, Callista Rinesmith, Cristobal S Vilches, Swati Chakarvarty

**Affiliations:** 1 Neuroscience, School of Behavioral and Brain Sciences, University of Texas at Dallas, Richardson, USA; 2 Neurophysiology, Global Innervation LLC, Dallas, USA; 3 Intraoperative Neuromonitoring Program, Labouré College of Healthcare, Milton, USA; 4 Neurology, NeuroCare.AI Academy, Dallas, USA

The Ethics Statement and Conflict of Interest Disclosures section has been updated to disclose the following:

**Financial relationships:** Shadab S. Kabir, Faisal R. Jahangiri, and Swati Chakarvarti declare(s) employment from Global Innervation LLC. Global Innervation LLC promotes and provides intraoperative neurophysiological monitoring.

